# Patient-specific arthroplasty guide in paediatric temporomandibular joint ankylosis management. An accuracy-based case series

**DOI:** 10.1186/s40902-026-00518-8

**Published:** 2026-07-06

**Authors:** Yehia El-Mahallawy, Nourhan M. Abdelmoneim, Heba Wagih Marei, Ibrahim Mohamed Abdelhamed

**Affiliations:** 1 Oral and Maxilofacial Surgery Department, Faculty of Dentistry, Alexandria University, Alexandria, Egypt; 2 Oral and Maxilofacial Surgery Department, Faculty of Dentistry, Fayoum University, Al Fayyum, Egypt

**Keywords:** TMJ ankylosis, Paediatrics, Gap arthroplasty, CAD/CAM, Surgical guide

## Abstract

**Aim:**

To evaluate the accuracy, safety, and short-term functional outcomes of a patient-specific arthroplasty guide for the management of paediatric Temporomandibular Joint Ankylosis (TMJ-A).

**Methods:**

A prospective case series study was conducted on 7 children with TMJ-A. Virtual surgical planning and CAD/CAM fabrication were used to design a patient-specific arthroplasty guide with narrower osteotomy slits (width 5 mm) to allow precise bone removal and improved condylar fit. Preoperative and postoperative tomographic scans were utilized to measure the procedural accuracy of the computer-assisted operation. Functional outcomes included complication rate and maximal interincisal opening (MIO).

**Results:**

All children achieved satisfactory intraoperative mouth opening (> 30 mm). No intraoperative injury to the skull base or neurovascular structures was encountered. A 2.36 ± 0.18° of SMA deviation was reported on the right side, and 1.90 ± 0.46° on the left SMA. Regarding the linear VRH, 1.57 ± 0.33 mm deviation was reported in the right side, and 0.95 ± 0.46 mm in the left ramus length. Good agreement between planned and postoperative measurements for both parameters. Temporary facial nerve weakness occurred in 1 patient, which resolved within 8 weeks.

**Conclusion:**

Patient-specific arthroplasty guide with direction-dictating slits allowed accurate and safe gap arthroplasty in paediatric cohort, facilitating stable intraoperative and early postoperative outcomes. This technique may reduce surgical variability and improve safety in paediatric TMJ surgery.

**Trial Registration:**

Retrospective registration was performed at clinicaltrials.gov [*NCT07320911*/2026-1-6].

**Supplementary Information:**

The online version contains supplementary material available at 10.1186/s40902-026-00518-8.

## Introduction

Temporomandibular Joint Ankylosis (TMJ-A) is an incapacitating dentofacial deformity that impairs mandibular growth, restricts mouth opening, and leads to significant functional, esthetic, and psychosocial challenges. While Surgical intervention is inevitable, TMJ-A management in paediatric patients has its own complexities, particularly the risk of injury to adjacent anatomical structures (1). TMJ-A surgical intervention involves freeing the ankylotic bone mass in a gap arthroplasty procedure. Intuitive gauging of the resected mass could lead to re-ankylosis if insufficiently resected, or telescoping of the ramus and posterior face pillar, if over-resected [1, 2]. Furthermore, the proximity of the ankylotic mass to the middle cranial fossa and neurovascular structures makes paediatric patients vulnerable to iatrogenic injury owing to their apparent anatomy [3, 4].

The utilization of contemporary virtual planning and 3D-printing in the management of TMJ-A improves the reproducibility, reduces the intraoperative guesswork, and allows the execution with greater precision [1, 5]. To date, few reports have described computer-guided gap arthroplasty in a paediatric cohort. This technical description report aims to depict the utilization of virtual surgical planning for the management of paediatric TMJ-A. This pilot study evaluates the feasibility, accuracy, and early outcomes of this approach, hypothesizing that the combination of narrow-slit direction-dictating surgical guides and piezoelectric osteotomy provides safe and reproducible results in paediatric TMJ ankylosis.

## Patients and methods

### Selection of patients

This prospective pilot trial was conducted on paediatric patients (less than 15 years of age) with type III-A3/ IV-A4 intra-articular bony ankylosis of the TMJ, recruited from the period of October 2024 to October 2025 from the Alexandria University Teaching Hospital outpatient clinic. Patients with fibrous ankylosis (type I-A1/II-A2) were excluded from this trial, along with those with associated craniofacial syndromes [6, 7]. This case series was conducted based on the PROCESS guidelines [8]. The study was conducted in adherence to the Helsinki guidelines, and after approval from the local Research Ethics Committee (IRB: 1194–00010556). Written informed consent was obtained from all participants’ legal guardians regarding their participation in the study after clarifying the nature of the operation and the utilization of their medical records after de-identification.

### Virtual surgical planning

**Fig. 1:**
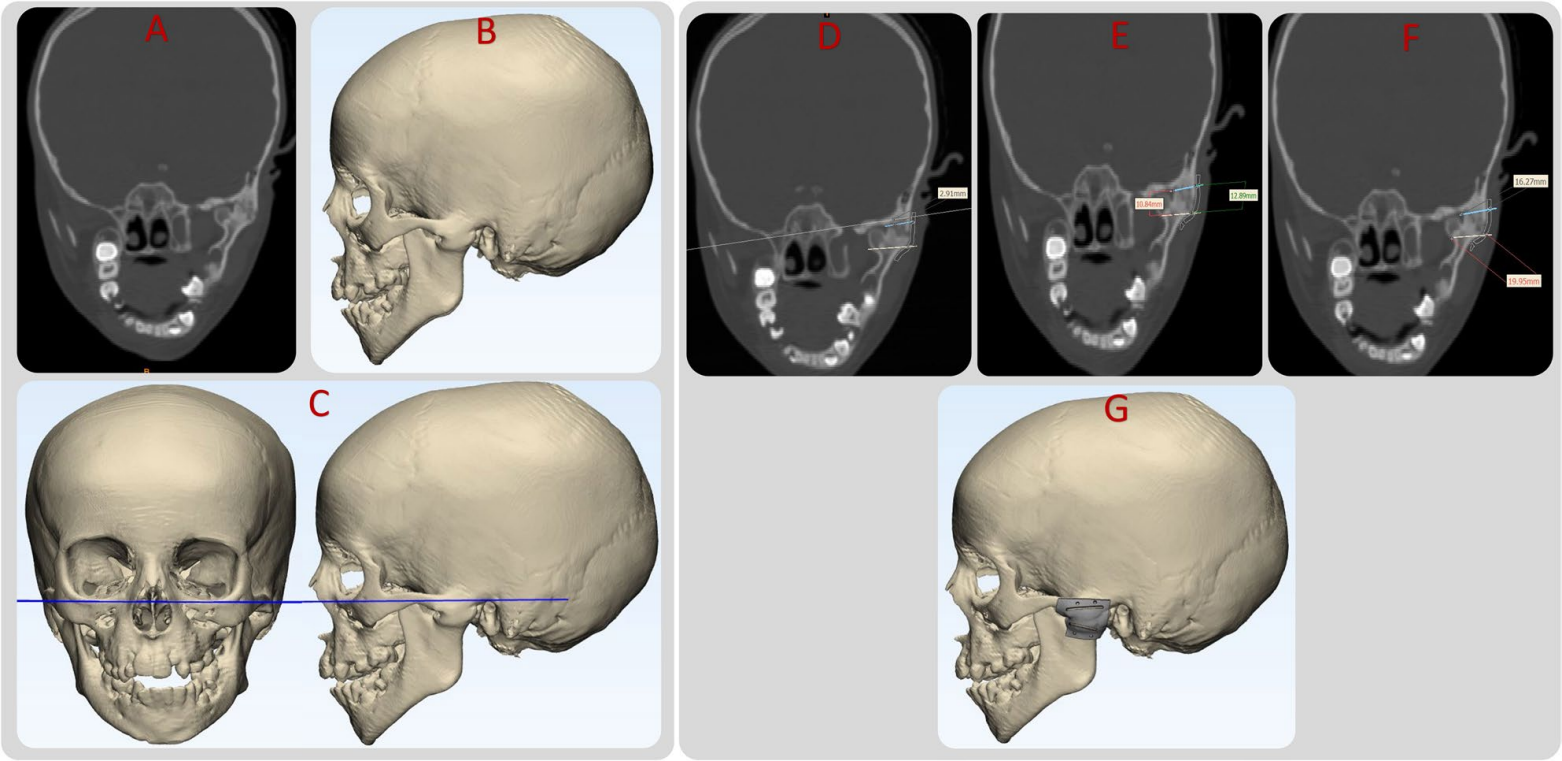
An illustrative description of the virtual planning methodology utilized for the design of the arthroplasty guide. **A**, a coronal MSCT scan cut showing ankylosis in the left TMJ. Note the improper orientation owing to the difficulty in child orientation during CBCT acquisition. **B**, Segmentation of the MSCT to create an ankylotic craniofcial 3D model. **C**, creation of the reorientation plane based on the Frankfort horizontal plane. **D**, the selected cranial and condylar osteotomies were checked on the MSCT scan with relation to the FHP. **E**,** F**, the depth of both osteotomies was measured and recorded for the surgery. **G**, Arthroplasty guide was designed on the 3D craniofacial 3D model, with a slot design based on the Piezotome US25 insert profile thickness. Two cranial and 2 cudal mini screw bore holes were designed for guide fixation

Multi-Detector Computed Tomography (MDCT) (Philips Brilliance 64 MDCT, Philips, Eindhoven, Netherlands) scans were performed for each patient, and the DICOM files were processed through segmentation software to produce a three-dimensional reconstruction of the craniofacial skeleton. (Mimics 19.0, Materialise, Leuven, Belgium). The 3D craniofacial structure could not be separated in the segmentation software owing to the ankylotic mass (Fig. 1).

The segmented model was transferred to a 3D-planning software (3Matic software, Materialise), where the Frankfurt Horizontal Plane (FHP) orientation was created through the superior rim of the external acoustic meatus and the left infra-orbital rim. This was created to orient any osteotomies in relation to the cranial base. A condylar, inferior, and a cranial, superior, resection plane are designed and spatially oriented. The position of both planes was verified on the MDCT, along with the FHP, to ensure the safety of the osteotomy spatial position, particularly the cranial osteotomy relation to the cranial base. The cephalocaudal distance between both resection planes was gauged from 10 mm to 15 mm according to each case to establish the optimal gap size, while preserving the ramus height [9].

The *arthroplasty guide* was designed on the lateral surface of the ankylotic mass with a 2 mm offset parameter. The guide was fitted on the anatomical landmark of the ankylotic mass, the ascending ramus, and the caudal and lateral surfaces of the zygomatic arch. The guide incorporated two narrow slits, one for the condylar and the cranial osteotomies. Each slit width was 0.5 mm, based on the thickness of the piezotome insert (US25, Woodpecker Medical Co Ltd, Guangxi, China). These slots constrained the osteotomy path, thereby reproducing the planned resection accurately during surgery. In addition, four 2.0-miniscrew fixation bore holes were incorporated in the *arthroplasty guide* design to allow temporary stabilization of the guide with screws. The depth of the proposed osteotomies was measured virtually from the outer surface of the guide to reduce the risk of injury to the neurovascular structures. This virtually determined value was gauged against the 17 mm working length of the piezo-US25 insert. This allows a direction-dictating design of the guide (Fig. 1).

The final design was exported as an STL file to a 3D-printing software (NETFAB, Autodesk, CA, USA). The *arthroplasty guide* was printed using FDM technology and PLA material. The guide was sterilized in a glutaraldehyde solution for 24 h before surgery, as per the CDC recommendation [10].

### Surgical technique

Owing to the limitation in mouth opening, general anaesthesia was administered via fiberoptic nasotracheal intubation. Following a routine surgical preparation and draping, a preauricular approach was utilized with a temporal hockey-stick extension, which provides ample exposure of the ankylotic mass while minimizing traction on the branches of the facial nerve. Careful soft tissue dissection was carried out to delineate the margins of the ankylotic block and its surrounding articulations.

**Fig. 2:**
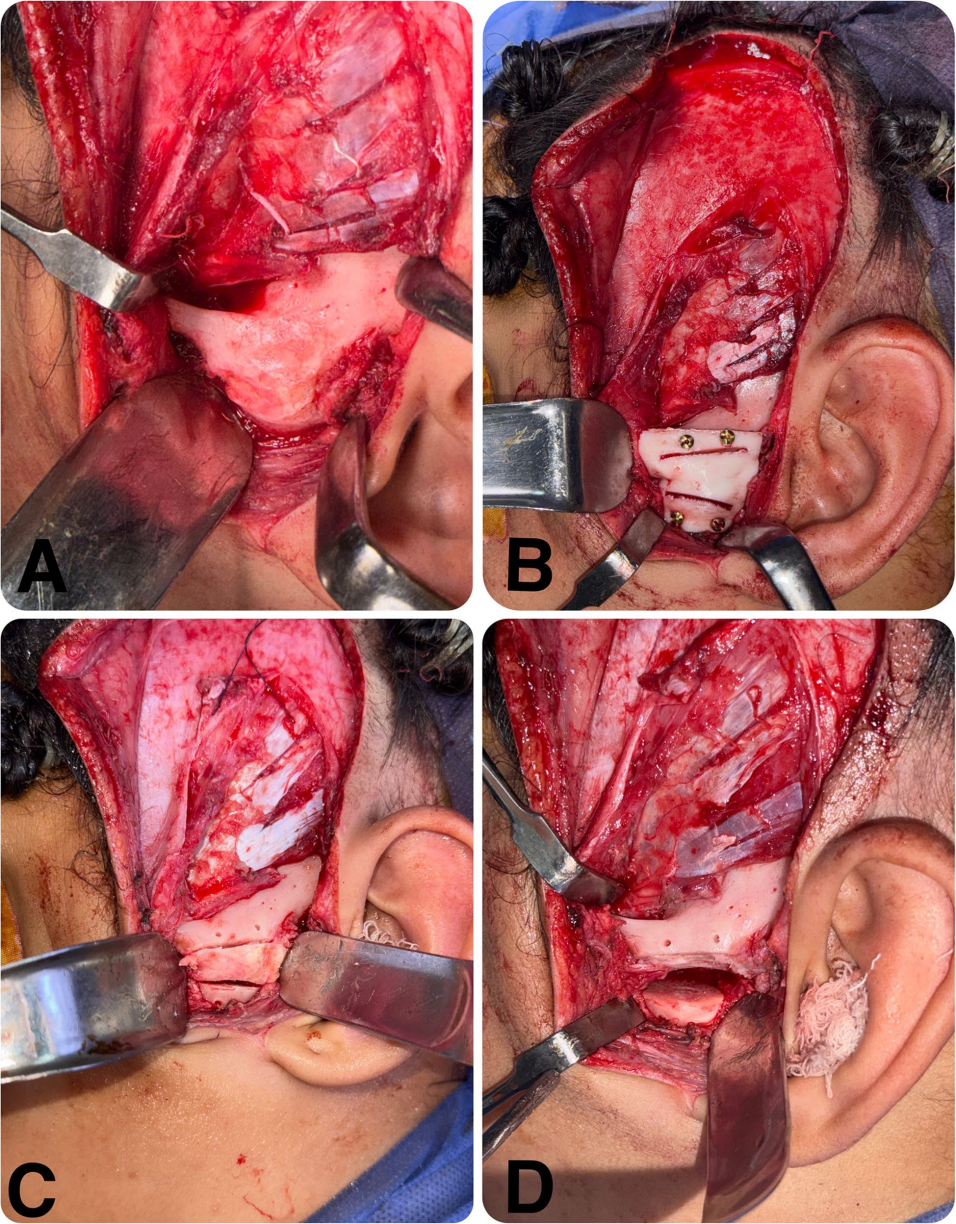
Application of the arthroplasty guide in the management of paediatric TMJ-A using. **A**, Surgical exposure of the ankylotic mass. **B**, Fixation of the arthroplasty guide on the ankylotic mass with the utilization of 4 mini-screws. **C**, Clinical illustration of the cranial and condylar osteotomies after guide removal. **D**, Gap after the removal of the ankylotic mass

The *arthroplasty guide* was positioned over the predetermined bony landmarks and stabilized with 2.0-mono-cortical screws (Fig. 2). Under copious saline irrigation, osteotomies were completed to the desired depth using a piezoelectric device on B-mode/ level 5–7 (Woodpecker) (Supplementary Fig. 1). The US25 insert was applied up to 1 mm of the predetermined depth in both the cranial and condylar slots. The remaining 1 mm was osteotomized on B-mode / level 3 till a drop is felt. Following this, the fixation screws were removed, and the guide was withdrawn. After guide removal, the anterior and posterior extensions of the osteotomies were completed. The ankylotic block was then mobilized along all its boundaries, and any remaining attachments were gently separated using fine osteotomes, minimizing the risk of injury to adjacent neurovascular structures (Fig. 2).

Following gap creation, mouth opening was assessed intraoperatively to determine the need for additional procedures. If the target of at least 35 mm maximal interincisal opening could not be achieved with gap arthroplasty alone, supplementary unilateral or bilateral coronoidectomy was performed. Interpositional material was placed when indicated. Finally, haemostasis was secured, a RediVac suction drain was inserted, and layered wound closure was performed.

### Postoperative management and physiotherapy

Patients were instructed in the use of cold compresses in the immediate postoperative period, followed by hot packs. Standard postoperative medications included antibiotics, anti-inflammatory drugs, and analgesics. An intensive physiotherapy program was initiated within the first 24 h after surgery [11]. Patients were instructed to perform repeated forced mouth-opening exercises using stacked wooden tongue depressors (tid / qid), gradually increasing the number of blades. This mechanical stretching was supplemented by prolonged gum chewing during the first postoperative month to promote active lateral and protrusive mandibular movement. Following the first postoperative month, patients continued the forced jaw-opening exercises (tid / qid) daily throughout the entire duration of the study.

### Postoperative clinical evaluation

Postoperative clinical assessment was conducted at 1, 4, 8, and 12 weeks. Wound healing complications were reported, including hematoma, infection, or facial nerve injury (House Brackmann-scale) [12]. Pain extent during physiotherapy was subjectively reported using the Visual Analogue Scale (VAS). The required period till the introduction of a solid diet was also reported for each of the enrolled patients. Objective Calliper gauging of the Maximal Interincisal Opening (MIO) was recorded for all of the enrolled patients, along with the degree of dental or chin midline deflection. The status of the occlusion was also monitored [13].

### Virtual surgical planning accuracy assessment

For the assessment of the procedural accuracy, an MDCT follow-up scan was obtained in the first postoperative week. Any detected remaining medial part of the ankylotic mass was outlined and reported. Assessment of the postoperative accuracy of the virtual design was conducted in a standard methodology as proposed by van Baar et al. and El-Mahallawy et al. (GOM-Inspect Pro 2019, Braunschweig, Germany) [14, 15]. Accuracy of the computer-assisted procedure in maintaining the rams profile was evaluated by angular and linear parameters, where the absolute mean (Δ) deviation was calculated by subtracting the actual postoperative values from the virtual preoperative ones. For the sake of reproducibility, a description of point location is presented in the *Supplementary Data*. These evaluated parameters were:

- *The Sagittal Mandibular Angle (SMA)*: Angular assessment of the gonial angle was calculated between the Vc-Hc line and the Vc-Ci line. The SMA was calculated for both the right and left gonial angles (*Supplementary Data*).
- *The Vertical Ramus Height (VRH)*: Linear assessment of the ramus height was calculated between the Vc point and the Frankfurt Horizontal Plane (FHP). The VRH was calculated for both the right and left ramus pillar (*Supplementary Data*).

### Statistical analysis

Descriptive analysis of the collected data was performed using IBM SPSS v.23.0 (IBM Corp, NY, USA). Data were presented in absolute mean, standard deviation, and range, with data significance set at the 5% level. A two-tailed ICC test was utilized to determine the degree of agreement between virtual preoperative and actual postoperative radiographic values.

## Results

**Fig. 3:**
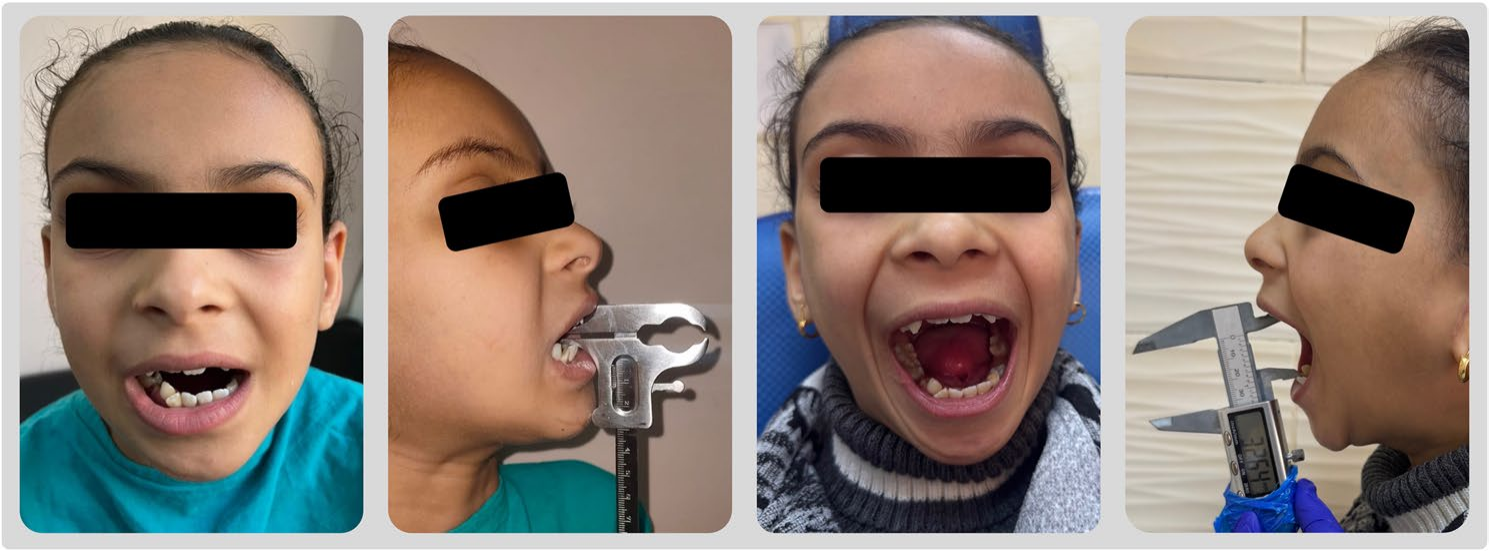
Preoperative and 3-month postoperative facial and side profile view of a paediatric patient

Seven patients (one male, six females) affected by intra-articular bony ankylosis of the TMJ were included in this study. All patients were below 15 years old at the time of surgery, with a mean age of 9.38 ± 1.3 years. Upon radiographic examination, 5 joints were of type III/ A3 ankylosis, while type IV/ A4 was reported in 2 joints. All of the enrolled cohort had a history of previous facial trauma, with a mean history of 21.25 ± 5.23 months from the time of surgery (Fig. 3).

The surgical approach was standardized in all of the enrolled patients, where the arthroplasty guide was appropriately adapted and secured intraoperatively. No remarkable bleeding was noticed intraoperatively, and procedural conduction was uneventful and safe. A temporalis muscle flap was utilized as an interpositional material in 3 joints, while no interpositional material was employed in the remaining joints. No clinical postoperative complication was observed in the follow-up interval. All patients reported grade I normal function in the HB-scale, except for one patient, who reported moderate limitation in eyebrow elevation (grade III). However, this was a transient impairment that resolved in the third follow-up session.

Preoperative mean MIO was 9.41 ± 4.73 mm, which posed a severe limitation in the mandibular kinematics. The mean achieved intraoperative values of MIO were 38.56 ± 1.91 mm. This was achieved by sole arthroplasty osteotomies in 5 cases, while an ipsilateral and bilateral coronoidectomy was required in one case each. The postoperative physiotherapy was started in all of the patients on the second postoperative day, with great emphasis on its importance to the parents. The mean recorded MIO in the first postoperative week was 34.13 ± 0.58 mm with a reported mean VAS score value of 6.86 ± 0.69. The achieved mouth opening was maintained across the follow-up period, with a mean value of 38.40 ± 1.74 mm, with a reported mean VAS score value of 0.57 ± 0.53.

**Fig. 4:**
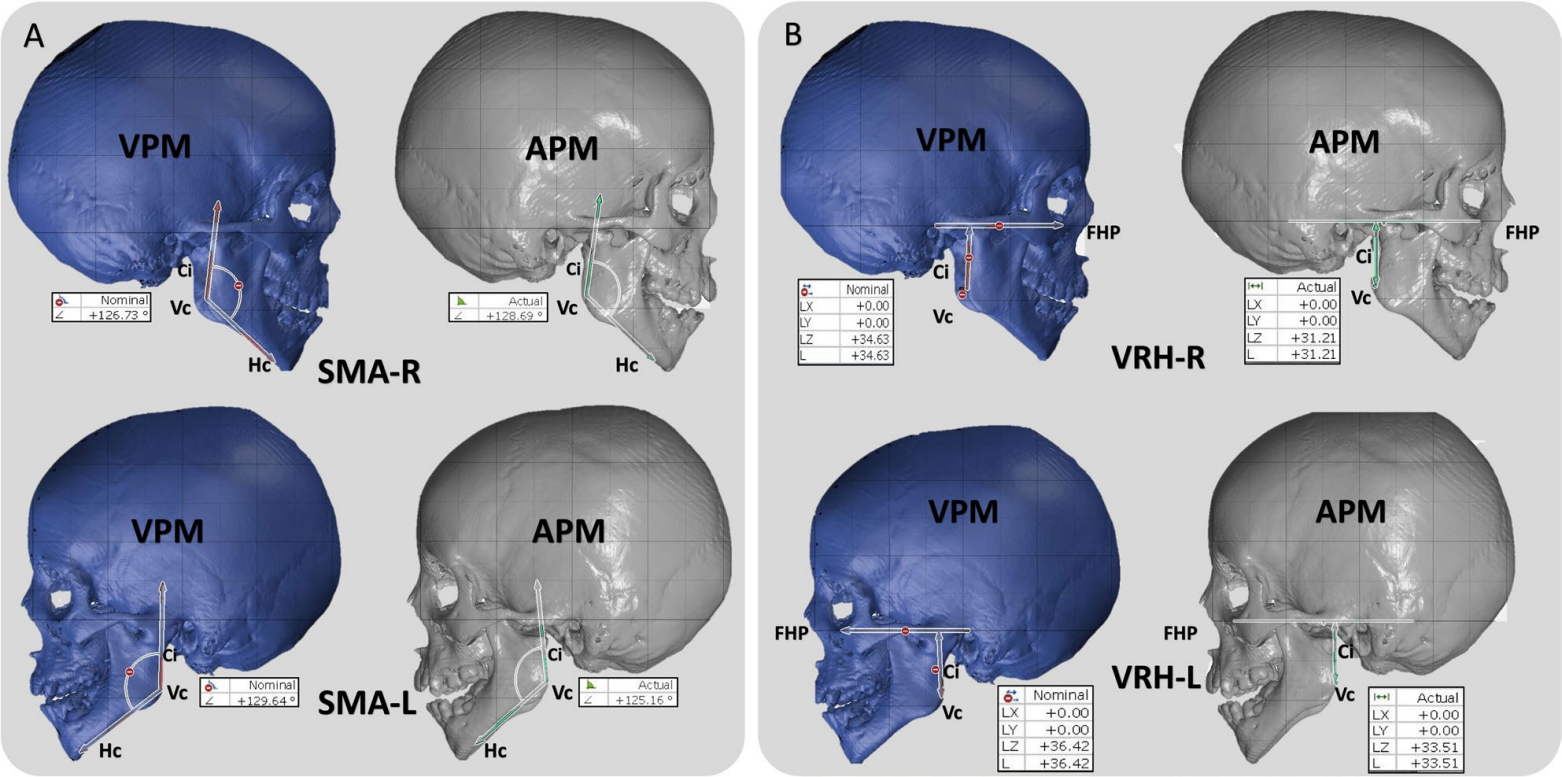
Demonstration of the Virtual Surgical Planning Accuracy Assessment outcome. **A**, Angular measurement assessment represented as the Sagittal Mandibular Angle. **B**, Linear measurement assessment represented as the Vertical Ramus Height. The models donated as VPM represent the Virtual Preoperative Model, and the APM represents the Actual Postoperative Model

Assessment of the virtual plan accuracy was conducted through the calculation of the virtual and actual SMA angular and VRH linear measurements. Table 1 demonstrates the angular assessment outcome of the SMA for all of the enrolled patients. A 2.36 ± 0.18° of SMA deviation was reported in the right side, and 1.90 ± 0.46° for the left SMA. A good degree of agreement between the actual postoperative and virtual preoperative SMA values for both the right (ICC = 0.807) and left angles (ICC = 0.847) (Fig. 4) (Table 1).

**Table 1:** Absolute mean deviation values for the virtual surgical planning accuracy assessment parameters (APM-VPM)

Δ	SMA / °		VRH / mm	
	*R*	L	*R*	L
‘|x̄|’ ± SD	2.36 ± 0.18	1.90 ± 0.46	1.57 ± 0.33	0.95 ± 0.46
Min - Max	2.16–2.65	1.19–2.36	1.23–2.04	0.13–1.43
ICC (95%-CI)	0.807 (0.333–0.954)	0.847 (0.452–0.964)	0.787 (0.299–0.951)	0.879 (0.553–0.971)
*P*	**0.005** ^*^	**0.002** ^*^	**0.004** ^*^	**< 0.001** ^*^

Δ: Deviation; ‘|x̄|’: Absloute mean; *SD* Standard Deviation, *APM* Actual Postoperative Model, *VPM* Virtual Preoperative Model, *SMA* Sagittal Mandibular Angle, *VRH* Vertical Ramus Height, *R* Right Side, *L* Left Side, *Min* Minimum value, *Max* Maximum Value, *ICC* Interclass Correlation Coefficient

*Statistically significant difference at *p* value ≤ 0.05

ICC Outcome Values: <0.5 Poor agreement, 0.5 to < 0.75 Moderate agreement, 0.75 to < 0.9 Good agreement, 0.9–1.0 Excellent agreement

Regarding the linear VRH, 1.57 ± 0.33 mm deviation was reported in the right side, and 0.95 ± 0.46 mm in the left ramus length. A good degree of agreement between the actual postoperative and virtual preoperative VRH values for both the right (ICC = 0.787) and left (ICC = 0.879) ramus lengths (Fig. 4) (Table 1). None of the patients demonstrated excessive shortening of the ramus or postoperative crossbite.

## Discussion

The surgical management of paediatric TMJ-A continues to be one of the most technically demanding areas of maxillofacial surgery. Surgeons are confronted by the need to create a reproducible and functional gap through irregular and dense ankylotic bone while working in close proximity to vital neurovascular structures. Inaccurate osteotomies risk both inadequate clearance, which predisposes to recurrence, or excessive bone removal, which can compromise ramus height [16].

In the present series, the use of patient-specific guides designed with narrow osteotomy slits allowed precise translation of the virtual plan to the operative field. The close anatomical fit improved the reproducibility of gap dimensions and constrained instrument trajectory, minimizing the risk of unintended cutting. The designed arthroplasty guide facilitated stable fixation and safe execution of the planned osteotomies through regular approaches.

The integration of piezoelectric devices for osteotomy represented an additional advance. Compared with rotary burs or oscillating saws, piezo-surgery provides controlled, selective cutting of mineralized tissue while sparing adjacent soft tissues. This is particularly advantageous in the paediatric craniofacial region, where safety margins are narrow, and structures such as the maxillary artery and dura are at risk. Although operative time with piezoelectric instruments was longer than with burs, the enhanced safety profile, reduced intraoperative bleeding, and precision in depth-restricted slots outweighed this limitation [17].

Intraoperatively, blood loss was minimal, and no postoperative hematomas were observed. One patient developed transient limitation in eyebrow elevation, consistent with temporary weakness of the temporal branch of the facial nerve, which resolved spontaneously within weeks. These temporary neuropraxias resolved spontaneously and are consistent with previously reported outcomes following preauricular approaches [12].

The direction-dictating design of the arthroplasty guide was augmented by the preoperative assessment of the osteotomy depth and matching this information with the piezo insert working length. This is reflected in the procedural safety and a thorough resection of the ankylotic mass from a medial-lateral perspective. This is noteworthy as inadequate medial release has been implicated in higher recurrence rates. None of the patients in this pilot series demonstrated re-ankylosis or postoperative limitation of mouth opening. We attribute this in part to the accurate, planned gaps facilitated by the guides, and in part to the strict regimen of early and continuous physiotherapy, which has been emphasized by others as a key determinant of long-term success.

The design process was based on the 3D-orientation of the craniofacial structure through the FHP. This was a trial to outmanoeuvre the difficulty in image acquisition in the paediatric cohort. Obtaining and the reproducibility of MDCT in children is an arduous procedure, with difficulty in child orientation owing to the subject’s undisciplined motion [18]. Furthermore, orienting the cranial osteotomy with the orientation of the FHP provided safe conduct of the arthroplasty procedure with minimal risk of iatrogenic cranial base perforation. The utilization of the FHP was imperative in both the designing and the accuracy evaluation phases of the virtual process. Mounir et al. demonstrated the utilization of computer-assisted planning for the management of adult patients with TMJ-A [1]. Their report demonstrated a favourable outcome and accurate procedural conduction, yet the standardization of the accuracy evaluation protocol is vague.

We opted for a standardized method and even a statistical approach for the accuracy assessment of the virtual surgical planning. The accuracy appraisal methodology avoids the hassle of superimposition and focuses on the absolute deviation of the actual postoperative values from the virtual preoperative ones in the form of angular and linear measurements [14, 15]. Several modifications were performed to suit the ankylotic craniofacial structure. A condylar-inferior point was utilized to avoid the apparent ankylosis mass morphology. Also, the FHP was utilized as the standard tenet for ramus height measurements.

Our results are consistent with recent literature validating the accuracy of computer-aided surgical templates in craniofacial procedures. Minimal discrepancies were observed between planned and achieved gap dimensions, echoing reports from Lu et al. and others that CAD/CAM-based guides can reliably transfer virtual plans into the operative setting [19, 20].

Regarding the ramus height analysis, a good degree of agreement was calculated for both TMJ sides, with the greatest mean deviation of 1.23–2.04 mm. Reda et al. reported an actual loss in VRH of 16 mm in a study of a guided arthroplasty procedure in an adult cohort [21]. This difference could be attributed to the measurement methodology. By tailoring the gap dimensions to the individual anatomy, as visualized on 3D models, the technique avoided the adverse consequences associated with overly large resections and excessive loss of ramus height. This finding supports the concept that precision, rather than excessive removal, is the critical factor in successful ankylosis surgery. None of the patients demonstrated excessive shortening of the ramus or postoperative crossbite.

Angular assessment of the SMA reported a good degree of agreement between the actual postoperative and virtual preoperative values for both the right (ICC = 0.807) and left angles (ICC = 0.847). Maintenance of the ramus profile in paediatric patients is of paramount importance as it affects the aesthetic profile and the overall appearance, and psychology of the child. Maintenance of the ramus height and profile could be a culpable factor for the decline in the incidence of occlusal discrepancies, apart from the evident chin deflection on opening. In paediatric TMJ-A, where gap arthroplasty typically aims for a resection width exceeding 10 to 15 mm, the observed linear deviation of approximately 1–1.5 mm represents a clinically negligible discrepancy. Similarly, the angular deviation of approximately 2° is unlikely to affect surgical outcomes, as such minor angulation differences do not meaningfully alter gap dimensions or increase the risk of recurrence or adjacent tissue injury.

The study could be limited by its exploratory pilot nature, small sample size, and lack of a concurrent control group. However, a cohort of 7 joints is consistent with the majority of the literature, especially in prospective trials. Furthermore, the relatively short follow-up inspection period in this study precludes any conclusions regarding long-term stability or recurrence. Yet this point is beyond the aim of the current report, which focuses on the virtual methodology, procedural condition, and accuracy appraisal of the computer-assisted TMJ-A management procedure. Operative time was also longer with piezo-surgery, a factor that may affect adoption in resource-constrained settings. Nevertheless, the proposed VSP workflow offers distinct advantages over conventional, freehand technique, including enhanced predictability of bone resection margins, precision replication of pre-surgical planning, and reduced risk of accidental damage to adjoining vital structures. Future controlled trials are warranted to directly compare these clinical benefits against established surgical techniques.

Nevertheless, this report suggests that combining the patient-specific arthroplasty guide with a direction-dictating slits design and the soft tissue-prudent piezoelectric technology is both a feasible and safe option, which may enhance surgical precision in the management of paediatric TMJ ankylosis. Patient-specific direction-dictating arthroplasty guides facilitated controlled osteotomy placement with constraining instrument trajectory and consistent ankylotic mass ablation.

## Supplementary Information

Supplementary Material 1. Supplementary Data. Definition of the Bony Landmark utilized in the Virtual Surgical Planning Accuracy Assessment.

Supplementary Material 2. Supplementary Fig. 1. Intraoperative image showing the Piezotome US25 insert placed through the slot created in the arthroplasty guide.

## Data Availability

All data generated or analysed during this study are included in this published article.

## Abbreviations

TMJ: Temporomandibular Joint
MDCT: Multi-Detector Computed Tomography
3D: Three-Dimensional
STL: Standard Tessellation Language
PLA: Polylactic Acid
HB: House-Brackmann
tid: 3 times daily
MIO: Maximal Interincisal Opening
VRH: Vertical Ramus Height
TMJA: Temporomandibular Joint Ankylosis
DICOM: Digital Imaging and Communications in Medicine
FHP: Frankfurt Horizontal Plane
FDM: Fused Deposition Modeling
CDC: Centers for Disease Control
VAS: Visual Analogue Score
qid: 4 timed daily
SMA: Sagittal Mandibular Angle
ICC: Intra-class correlation coefficient
