# Supplementary material for: Patient-specific arthroplasty guide in paediatric temporomandibular joint ankylosis management. An accuracy-based case series
Source: Maxillofac Plast Reconstr Surg. 2026 Jul 6;48(1):18. doi: 10.1186/s40902-026-00518-8 (PMC13346373; doi:10.1186/s40902-026-00518-8)
Supplement: Supplementary file 1 — Supplementary Material 1. Supplementary Data. Definition of the Bony Landmark utilized in the Virtual Surgical Planning Accuracy Assessment. [file 40902_2026_518_MOESM1_ESM.docx]

**Virtual Surgical Planning Accuracy Assessment.**

- ***Bony Landmark Definition***

**Vertical corner (Vc):** Point Centretd at the angles of the mandible as per Brwon et al ^^[[1]](#footnote-1)^*^.

**Horizontal corner (Vc):** Point Centretd at the the canine teeth as per Brwon et al ^^[[2]](#footnote-2)^*^.

**Condylar Inferior (Ci):** Point on the most inferior point of the condylar neck ^[[3]](#footnote-3)^**.

**Orbitale:** The lowest point of the left inferior orbital rim.

**Porion:** The uppermost point of the external auditory meatus.

**Frankfurt Horizontal Plane (FHP):** plane connecting the porion points with the orbitale, representing the true horizontal skull plane in its neutral resting position.

- ***Angular Measurement Definition***

**The Sagittal Mandibular Angle (SMA):** Angular assessment of the gonial angle was calculated between the Vc-Hc line and the Vc-Ci line. The SMA was calculated for both the right and left gonial angles.

- ***Linear Measurement Definition***

**The Vertical Ramus Height (VRH):** Linear assessment of the ramus height was calculated between the Vc point and the Frankfurt Horizontal Plane (FHP). The VRH was calculated for both the right and left ramus pillar.

1. * Brown JS, Barry C, Ho M, Shaw R. A new classification for mandibular defects after oncological resection. The Lancet Oncology. 2016;17:e23-e30. https://doi.org/10.1016/S1470-2045(15)00310-1. [↑](#footnote-ref-1)
2. [↑](#footnote-ref-2)
3. ** The choice of the Ci point was to avoid miss-measurments that could occur owing to the abberent mrphoplogy of the ankylotic mass if the conydlar posterior point was utulized. [↑](#footnote-ref-3)
